# Exploring potential association between flow instability and rupture in patients with matched-pairs of ruptured–unruptured intracranial aneurysms

**DOI:** 10.1186/s12938-016-0277-8

**Published:** 2016-12-28

**Authors:** Lijian Xu, Lixu Gu, Hao Liu

**Affiliations:** 1 Shanghai Jiao Tong University and Chiba University International Cooperative Research Center (SJTU-CU ICRC), School of Biomedical Engineering, Shanghai Jiao Tong University, 800 Dongchuan Road, Minhang district, Shanghai, People’s Republic of China; 2 Graduate School of Engineering, Chiba University, 1-33, Yayoi-cho, Inage-ku, Chiba-shi, Chiba Japan

**Keywords:** Cerebral aneurysm, Wall shear stress, Computational fluid dynamic, Middle cerebral artery, Anterior communicating artery

## Abstract

**Background:**

Patients with multiple intracranial aneurysms present a great challenge to the neurosurgeon, particularly when presenting with subarachnoid hemorrhage. Misjudgment may result in disastrous postoperative rebleeding from the untreated but true-ruptured lesion.

**Methods:**

In this study, computational fluid dynamic simulations of two matched-pairs of ruptured–unruptured cerebral aneurysms were performed to investigate the potential association between flow instability and aneurysm rupture. Two pairs of cerebral aneurysms from two patients were located in the middle cerebral artery and the anterior communicating artery respectively.

**Results:**

Our results demonstrated highly disturbed states of the blood flows in the ruptured aneurysms of the two patients with multiple aneurysms, which are characterized by remarked velocity and wall shear stress (WSS) fluctuations at late systole. The ruptured aneurysms exhibit obviously temporal intra-cycle WSS fluctuations rather than the unruptured aneurysms of the same patient. Cycle-to-cycle fluctuations are further observed in the ruptured aneurysms when the flow turns to decelerate.

**Conclusions:**

The obvious differences observed between matched-pairs of ruptured–unruptured aneurysms imply that flow instability may be a potential source correlating to aneurysm rupture.

## Background

Cerebral aneurysms are cerebrovascular protruding sacs that develop in specific cerebrovascular sites. The most severe consequence of cerebral aneurysm is its rupture and intracranial subarachnoid hemorrhage (SAH), causing a high mortality rate. Multiple intracranial aneurysms are observed in 15–35% of patients with aneurysms who present with SAH [1–3]. For SAH patients with multiple aneurysms, it is very important for a medical team to determine which one causes SAH. Misdiagnosis is dangerous because the untreated but ruptured aneurysm may re-rupture soon [4, 5]. For patients without SAH, it is also vital to predict which aneurysms are prone to rupture and whether an operation is necessary or not. However, prediction of rupture status in patients with multiple cerebral aneurysms remains challenging for clinicians [6, 7].

Hemodynamic factors, particularly the wall shear stress (WSS)-related ones, play an important role in understanding the initiation, growth and rupture of cerebral aneurysms. The initiation of cerebral aneurysms is cautiously correlated with high WSS [8]. However, both high-WSS and low-WSS theories have been proposed to explain the growth and rupture of cerebral aneurysms [9–11]. Xiang et al. [9] carried out a statistical analysis on 38 ruptured and 81 unruptured cerebral aneurysms and pointed out that the low WSS and a high oscillatory shear index (OSI) might be correlated with aneurysm rupture. In the contrast, Cebral et al. [10] proposed a correlation on the elevated levels of the maximum WSS with aneurysm rupture based on an investigation of 210 cerebral aneurysms. There obviously exists a controversy between the high and low WSS-based theories, which may be due to the complicated rupture mechanisms as well as different experimental designs including limited sample size, inconsistent parameter definitions and simplified model assumptions. Recently, studies of flow instability associated with aneurysms have been carried out experimentally and numerically as a potential source correlating with the aneurysm rupture mechanism [12–18]. Baek et al. [15] investigated the flow instabilities and oscillatory WSS behavior of three patient-specific aneurysms of the internal carotid artery (ICA) and demonstrated that the presence of low-frequency-fluctuations were geometry-dependent because the aneurysm models showed pronounced fluctuations at different Reynolds numbers. Ford et al. [16] investigated the flow instabilities and confirmed the feature of high-frequency-fluctuations in all four idealized basilar tip models and two of the four patient-specific terminal basilar tip aneurysm models. Valen-Sendstad et al. [17, 18] reported the high-frequency flow fluctuation in certain terminal aneurysms and pointed out that it could also take place in the sidewall aneurysms if a siphon would be included in the geometry.

In this study, using computational fluid dynamic (CFD) simulations we aim at exploring the association between flow instability and aneurysm rupture in two patient-specific models with two matched-pairs of cerebral aneurysms, in which one is observed ruptured but the other remains unruptured. Through such comparative study we will be able to remove the uncertainties due to different individual models and to figure out how the feature of flow instabilities correlates with the aneurysm rupture in the same patient with multiple aneurysms.

## Methods

### Geometric modeling

**Fig. 1:**
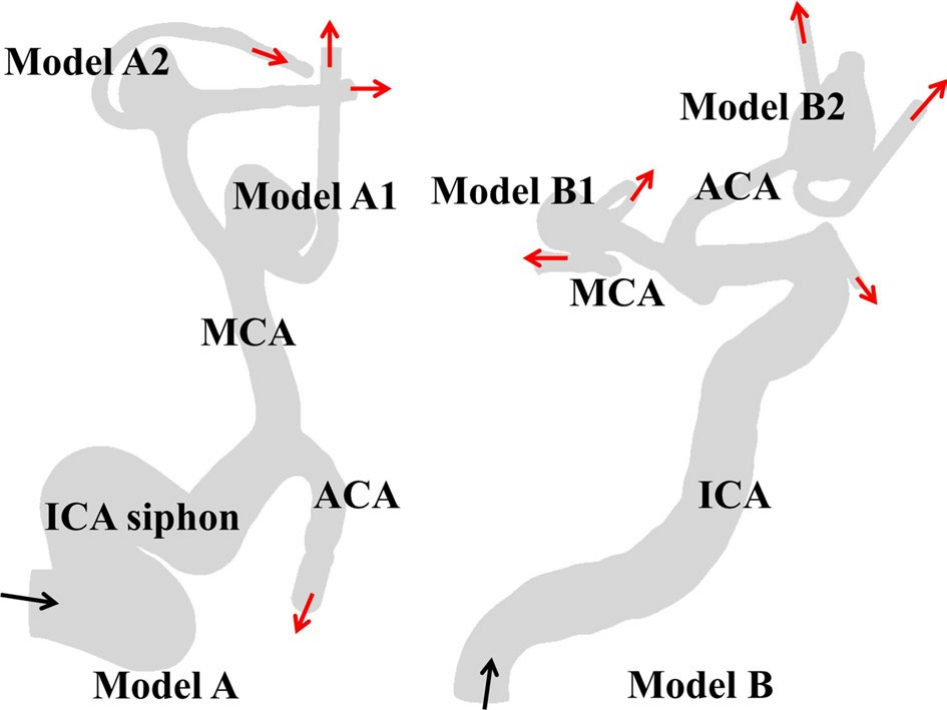
Four patient-specific cerebral aneurysm models in two pairs employed in simulations (unruptured aneurysms* A1* and* B1*, ruptured aneurysms* A2* and* B2*). *Black* and *red arrows* point to single inlets and multiple outlets, respectively

We focused on the multiple cerebral aneurysms with different rupture status in the same patient from the open Aneurisk database [19]. The matched-pairs of ruptured–unruptured aneurysms in the present study could be a reasonable comparative model for exploring potential indicator for aneurysm rupture since sharing the same inflow boundary conditions for the paired aneurysms in one patient could help removing the uncertainties due to individual differences. All the aneurysm models from the repository were reviewed for minimize selection bias. The register was approved by the local ethics committee and the informed consent was obtained for the use of imaging and clinical data from the involved patients. We obtained the images of four cerebral aneurysms before the occurrence of rupture from two patients as shown in Fig. 1. The first patient developed two aneurysms in the middle cerebral artery (MCA). One is located in the upstream MCA and the other is downstream with a short distance. The second patient had two aneurysms located in the anterior communicating artery (ACA) and MCA respectively. The following geometric parameters were calculated: maximum aneurysm diameter (D_max_); mean diameter of the ostium plane (D_ostium_); aspect ratio between aneurysm height and ostium diameter; and size ratio defined as the sac maximum height over the parent vasculature diameter. Details of demographic and geometric parameters of the aneurysms are summarized in Table 1. The patient-specific anatomic models of the cerebral aneurysms and vessels were extracted using the gradient-based level set segmentation algorithm from the open-source vascular modeling toolkit (VMTK) [20] and were further smoothened by the VMTK’s Taubin filter. We included the vessel geometry features as much as possible and extended the outlets by ten diameters of each vessel at outlet to reduce the boundary artifacts. The reconstructed surface files were finally imported into the ANSYS ICEM 15.0 to be meshed.

**Table 1:** Demographic and geometric parameters of the aneurysms

Model	Age	Sex	Location	Status	D_max_ (mm)	D_ostium_ (mm)	Aspect ratio	Size ratio
A1	77	F	MCA	Unruptured	6.43	5.24	0.97	2.57
A2	77	F	MCA	Ruptured	4.80	4.46	0.78	2.38
B1	71	M	MCA	Unruptured	6.81	5.48	0.93	2.92
B2	71	M	ACA	Ruptured	8.69	4.68	1.69	5.93

### Boundary conditions

**Fig. 2:**
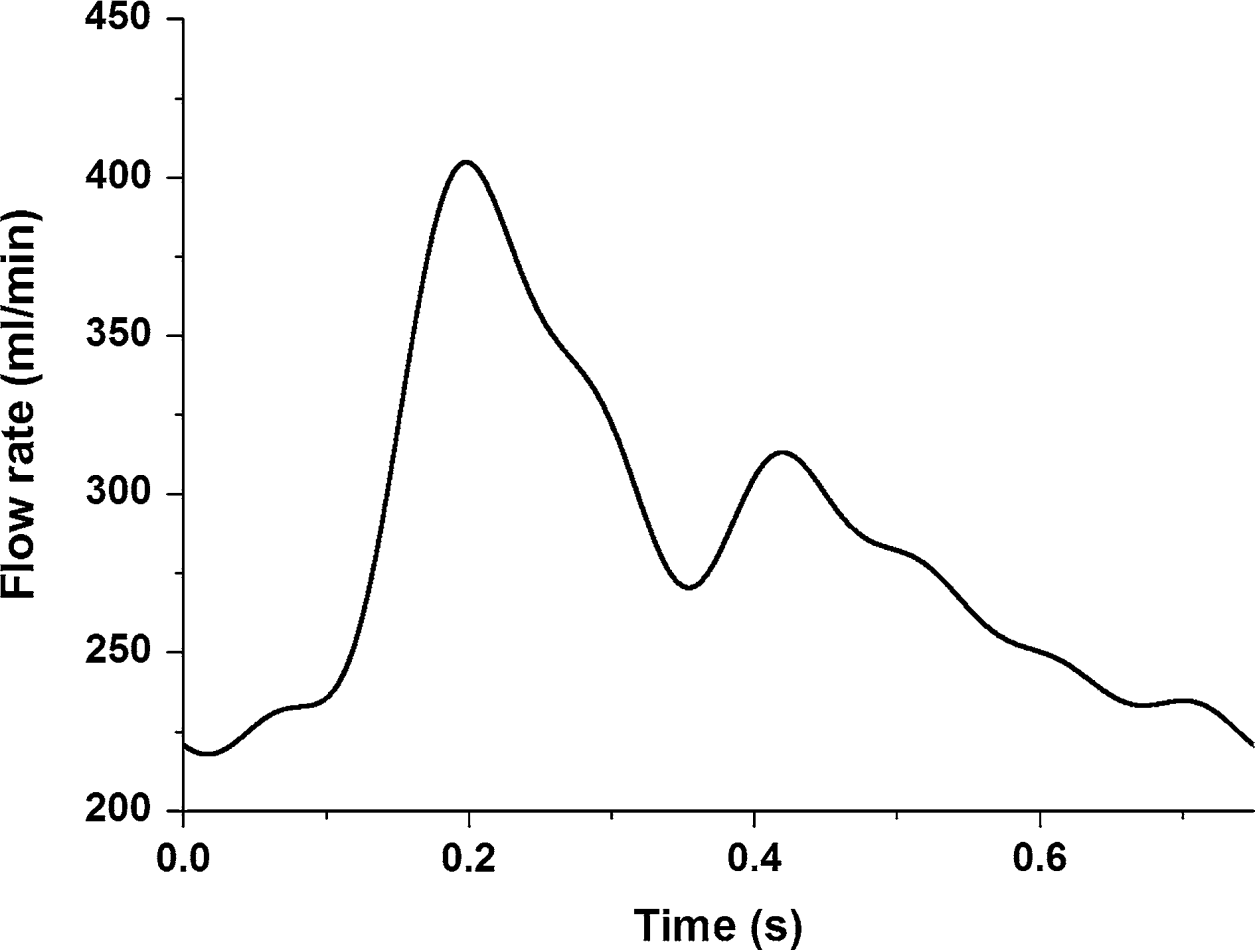
The inlet flow rate waveform over a complete cardiac cycle of model A with an average velocity of 0.31 m/s

At the inlet we prescribed fully developed Womersley velocity profiles based on a flow rate waveform obtained from [15] such as

$$ u\left( {r,t} \right) = u_{0} \left[ {1 - \left( {\frac{r}{R}} \right)^{2} } \right]\left\{ {1 + A \sum \limits_{n = 1}^{N} ( a_{n} \cos (n\omega t) + b_{n} \sin (n\omega t))} \right\} $$

where the constant term *u*_*0*_ represents the average velocity, *R* the vessel’s radius, *A* the amplitude factor (*A* = 1), *N* the number of harmonics (*N* = 7), and ω the angular frequency. The pairs of *a*_*n*_ and *b*_*n*_ normalized by constant mode *u*_*0*_ are calculated to be (−0.152, 0.129), (−0.111, −0.031), (0.043, −0.086), (0.028, 0.028), (0.002, 0.010), (−0.027, 0.012), (−0.0005, −0.026) corresponding with the seven harmonics. A heart rate of 80 beats per minute was used, leading to cardiac cycle duration of 0.75 s. An average inflow velocity of 0.31 m/s was applied at the inlet assuming that flow rate scales with cross-sectional area as shown in Fig. 2. The Reynolds number is defined as *Re* = *uD/v*, where *u* denotes the inflow mean velocity, *D* the vessel’s diameter, and *v* the kinematic viscosity of the fluid. The Womersley number is given by $R\sqrt {\omega /v}$. The blood density and kinematic viscosity is 1025 kg/m^3^ and 3.5 × 10^−6^ m^2^/s, respectively. The average Reynolds numbers of models A and B are accordingly 354 and 397, respectively, which correspond with two Womersley numbers of 3.23 and 3.47, respectively. At outlets zero pressures and zero velocity-gradients were imposed. On vessel surfaces we assumed that the compliant wall-induced deformation is negligible and hereby employed the non-slip conditions.

### Computational fluid dynamic modeling

Blood is modeled as an incompressible and Newtonian fluid. The governing equations for the fluid are unsteady and incompressible three-dimensional Navier–Stokes equations.

1 $$ \frac{{\partial u_{i} }}{{\partial x_{i} }} = 0 $$

2 $$ \frac{{\partial u_{i} }}{\partial t} + u_{j} \frac{{\partial u_{i} }}{{\partial x_{j} }} = - \frac{1}{\rho }\frac{\partial P}{{\partial x_{i} }} + v\frac{{\partial^{2} u_{i} }}{{\partial x_{j}^{2} }} $$

where *u*_*i*_ is the velocity component in the direction *x*_*i*_ (*i, j* = 1, 2, 3), *P* the pressure. Transient flow simulations were performed using CFX 15 (ANSYS), which utilizes an element-based finite volume approach to discretize in space, and a high-resolution scheme for the stabilization of the convective term. Time discretization is achieved by the second-order backward Euler scheme. Tri-linear finite element based functions are used as interpolation scheme. CFX uses an implicit coupled solver, in which all the hydrodynamic equations are solved as a single system. Non-linear equations are linearized, which are then solved by an algebraic multigrid (AMG) solver. The convergence criteria for iterative errors were set to be of 10^−4^. A fixed time step is used to facilitate post-processing and comparison. No turbulence model was applied due to the relatively low Reynolds number in this study. The simulations required approximately 44 h of CPU time on a PC with an Intel Xeon (2.9 GHz); parallel processing on 32 nodes was performed using Platform MPI. All the simulations were conducted up to nine cardiac cycles and the computed results of the last six cycles were used for further analysis.

### Mesh independence

In order to accurately calculate the WSS at near-wall regions, the grids are clustered sufficiently to the body wall so as to resolve the boundary layer as well as vortical flow adjacent to the vessel surface. In the present study, three prism layers were utilized to solve near-wall regions with an average nodal space increasing by 1.2. The distance of the first layer to the vessel surface was fixed at 0.02 mm. Tetrahedral elements were generated for the remainder of the lumen with a minimum element size of 0.025 mm and maximum element size of 0.1 mm. The total numbers of elements ranged from 8 to 16 million elements in different aneurysm models. Mesh-dependence was studied together with the time increment effect to confirm high spatial and temporal resolutions in predicting flow instability in cerebral aneurysms. Three time increments of 0.1, 0.25, and 0.5 ms were taken for comparison and the results with the time step of 0.25 and 0.1 ms agreed reasonably each other, capable of capturing high-frequency flow instability. Furthermore, the grid refinement was investigated with a minimum mesh size of 0.03, 0.025, and 0.01 mm, and together with the time increment refinement. A mesh system with a minimum element size of 0.025 mm and a time step 0.25 ms was confirmed to be capable to provide sufficiently high resolution of the flow instability in the present cerebral aneurysms models and were substantially employed in all the simulations. To assess whether the flow field was properly resolved, the spatial and temporal resolutions were assessed by computing the viscous length *l* ^+^ and time scale *t* ^+^, such as

3 $$ l^{ + } = \frac{{u_{*} d_{x} }}{v} , $$

4 $$ t^{ + } = \frac{{u_{*}^{2} d_{t} }}{v}, $$

5 $$ u_{*} = \sqrt {vs_{ij} } ,  s_{ij} = 0.5 \left( {\frac{{\partial u_{i} }}{{\partial x_{j} }} +  \frac{{\partial u_{j} }}{{\partial x_{i} }}} \right), $$

where u_*_ is the friction velocity at the vessel wall, *d*_*x*_ the maximum element size, *d*_*t*_ the time step, *s*_*ij*_ the fluctuating component of strain rate. In the present simulations, *l* ^+^ and *t* ^+^ are chosen to be on the order of 1.0. We did not attempt to fully resolve the smallest temporal or spatial scales, but rather the scales with the major energy. High-resolution simulations were performed to minimize artificial diffusion and capture the correct flow states.

### Shear forces and kinetic energy

Based on the computed spatio-temporal WSS distributions, OSI was calculated to describe the oscillatory feature of the WSS vector, such as

6 $$ OSI = 0.5 \left( {1 - \frac{{\left| {\mathop \smallint \nolimits_{0}^{T} \overline{WSS} dt} \right|}}{{\mathop \smallint \nolimits_{0}^{T} \left| {\overline{WSS} } \right|dt}} } \right) , $$

where 0 < *OSI* < 0.5 with a value of 0 being no variation of the WSS vector, and of 0.5 being 180° deflection of the WSS direction.

To assess the fluctuating kinetic energy (FKE) we further made decomposition of the instantaneous velocity *u*_*i*_(*x*, *t*) with a mean $\overline{{u_{i} }} (x,t)$ and a fluctuating component $u_{i}^{{\prime }} \left( {x,t} \right)$ (*i* = 1, 2, 3), so that

7 $$ u_{i} (x,t) = \bar{u}_{i} (x,t) + u_{i}^{\prime } (x,t) $$

Thus the FKE can be defined as,

8 $$ FKE\left( {x,t} \right) = \frac{1}{2} < u_{i}^{{\prime }} (x,t) \cdot u_{i}^{{\prime }} (x,t) > $$

## Results

### Flow patterns and hemodynamic factors

**Fig. 3:**
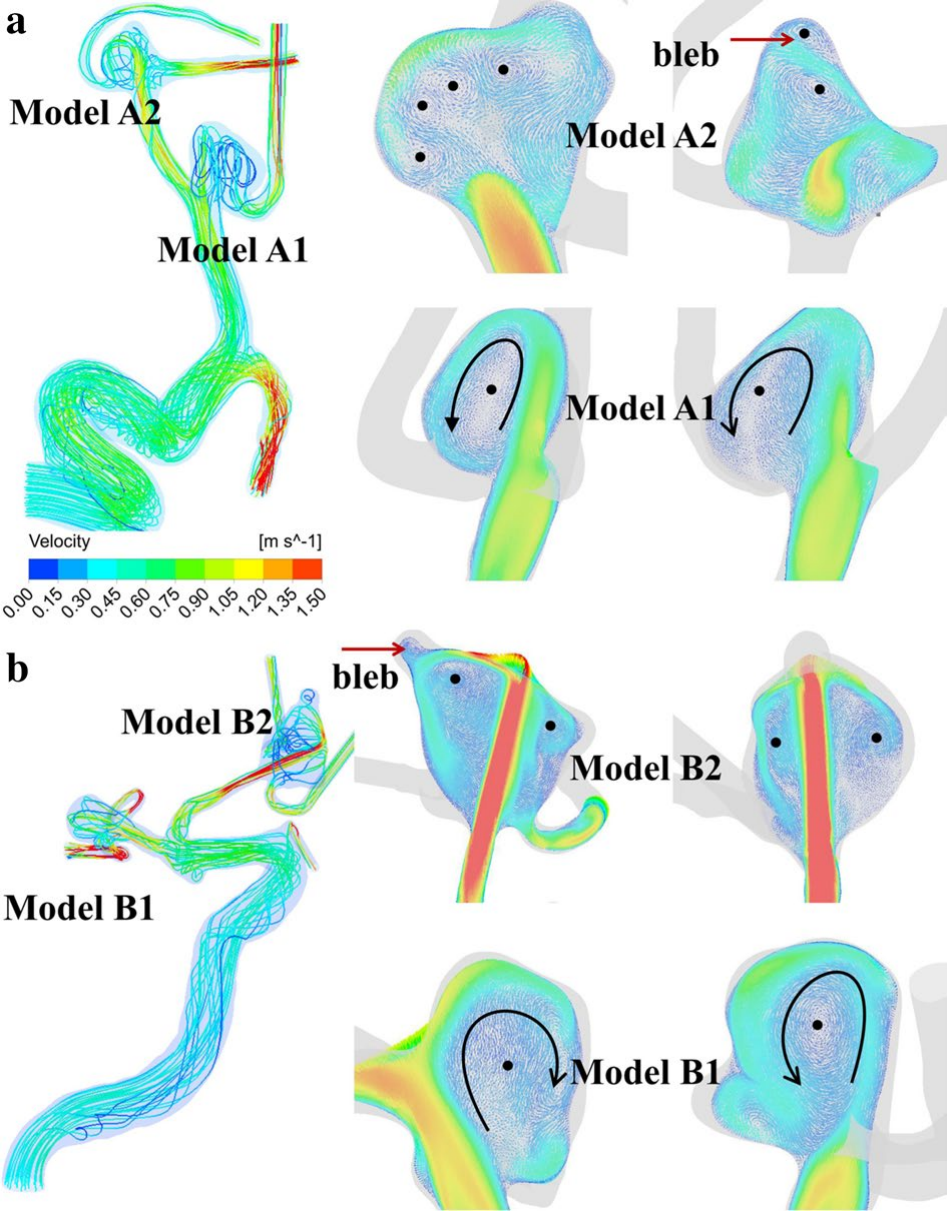
Visualized flow patterns at peak systole in two cutting planes with a 40% opaque surface of model A (**a**) and model B (**b**). The *black circles* correspond to vortex cores and the *red arrows* point to the recirculating zones with slow flow due to flow separation at the blebs. The two ruptured aneurysms (models *A2* & *B2*) present a feature of complex unstable flow patterns with multiple vortices and one recirculation zone at the blebs, while models *A1* and *B1* (unruptured) show relatively simple stable flows with a single vortex

**Fig. 4:**
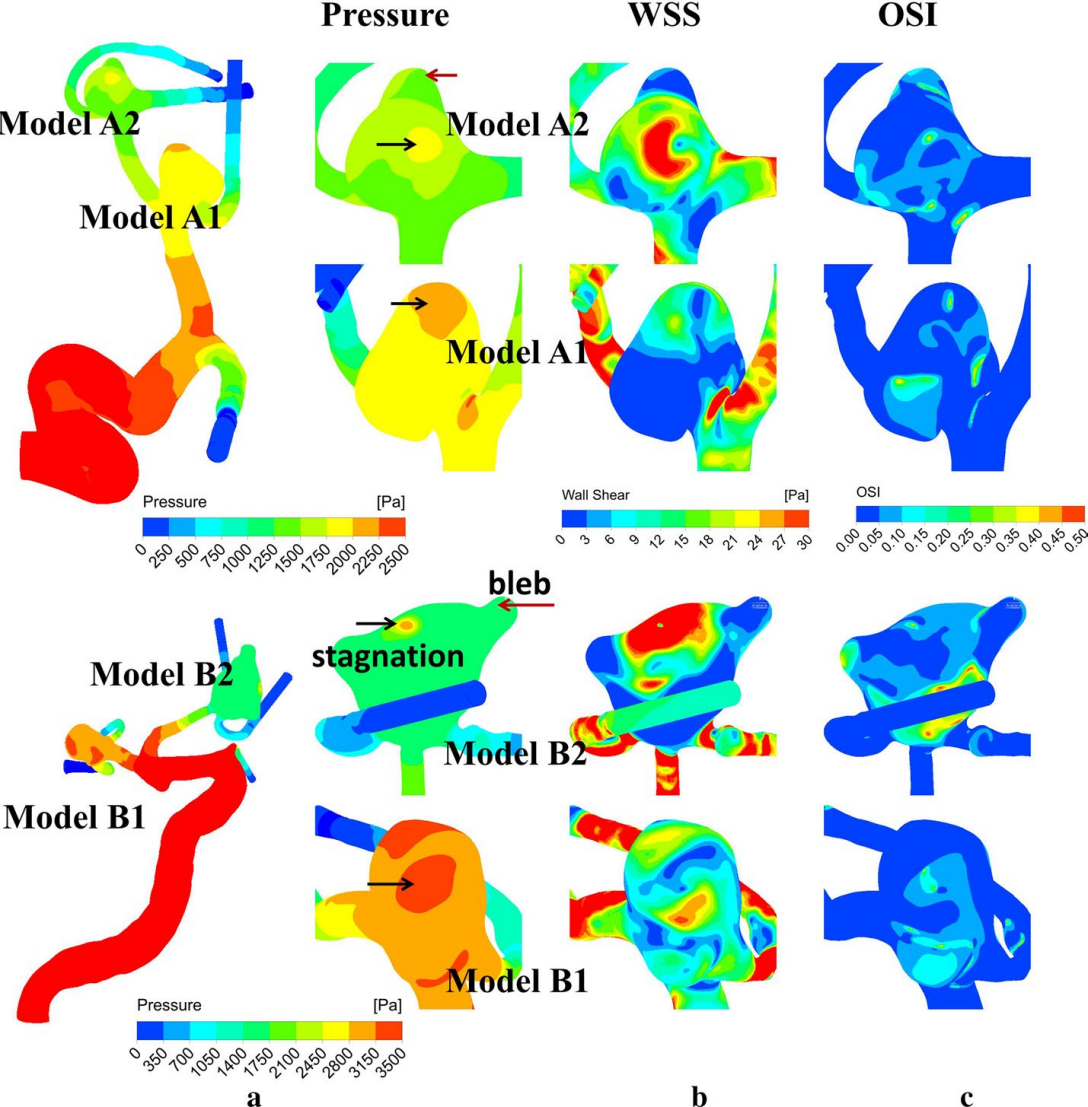
Distributions of pressures (**a**), WSSs (**b**) and OSIs (**c**) at peak systole for model* A* (*upper*) and model* B* (*lower*). *Red* and *black arrows* point to the recirculating zones at the blebs and the stagnation regions, respectively. There obviously exists a featured distribution of high OSIs and low WSSs at the blebs, with high pressures and WSS gradients in the two ruptured aneurysms* A2 * and* B2*

Figure 3 illustrates the streamline and velocity distributions in two multiple cerebral aneurysm models. It is seen that the blood flows inject into the aneurysm at a specific angle from the parent vessel, leading to one or multiple vortices inside the aneurysm. Models A1 and B1 (unruptured) obviously show relatively stable flows with a single vortex whereas models A2 and B2 (ruptured) present a complex feature of unstable and disturbed flow patterns with multiple vortices and one recirculation zone at the blebs (red arrows). Figure 4 shows the pressure distributions around stagnation regions (black arrows) are at a very high level, which leads to adverse local pressure gradients against the flow into the aneurysm. The local stagnation regions inside the aneurysm obviously correspond to low WSSs and high OSIs surrounded by high WSS regions. The ruptured aneurysms A2 and B2 are exposed to high OSI and low WSS at the blebs, corresponding with high pressures and steep spatial WSS gradients in the stagnation regions.

### Flow fluctuations and fluctuating kinetic energy (FKE)

**Fig. 5:**
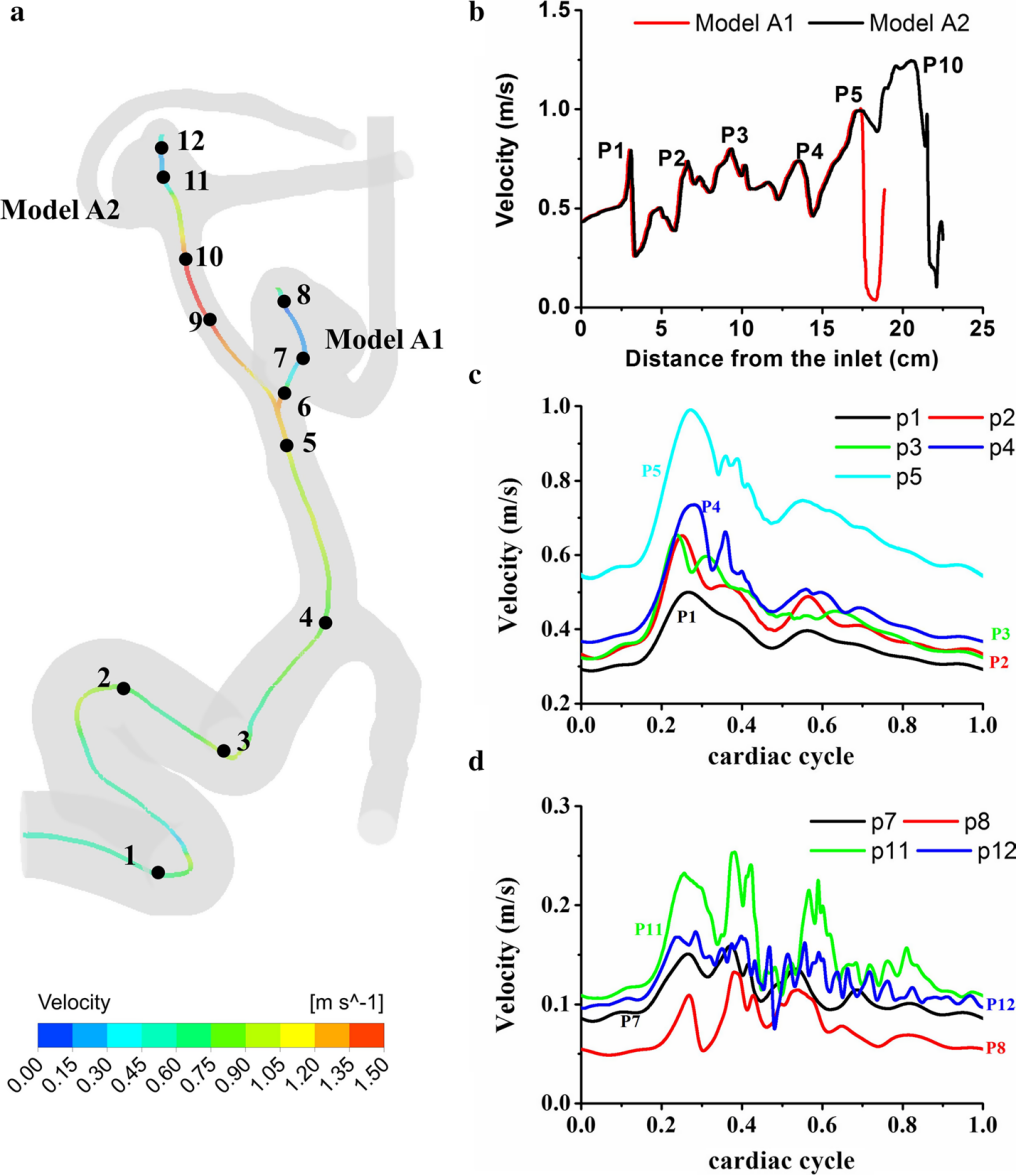
Velocity distributions aligning with centerlines at peak systole (**a**, **b**) and time-varying velocities at feature points for model* A* (**c**, **d**). Complicated velocity profiles with multiple inflection points are found at the bends of ICA siphon (Points* 1*–*5*). Velocity fluctuations are observed in the upstream arteries and aneurysm sacs. Note that the rupture aneurysm* A2* (Points* 11*,* 12*) exhibit stronger velocity fluctuations than unruptured aneurysm* A1* (Points* 7*,* 8*). The peak Reynolds numbers of the upstream parent arteries of aneurysms are calculated to be 627 and 554 for models* A1* and* A2*, respectively

**Fig. 6:**
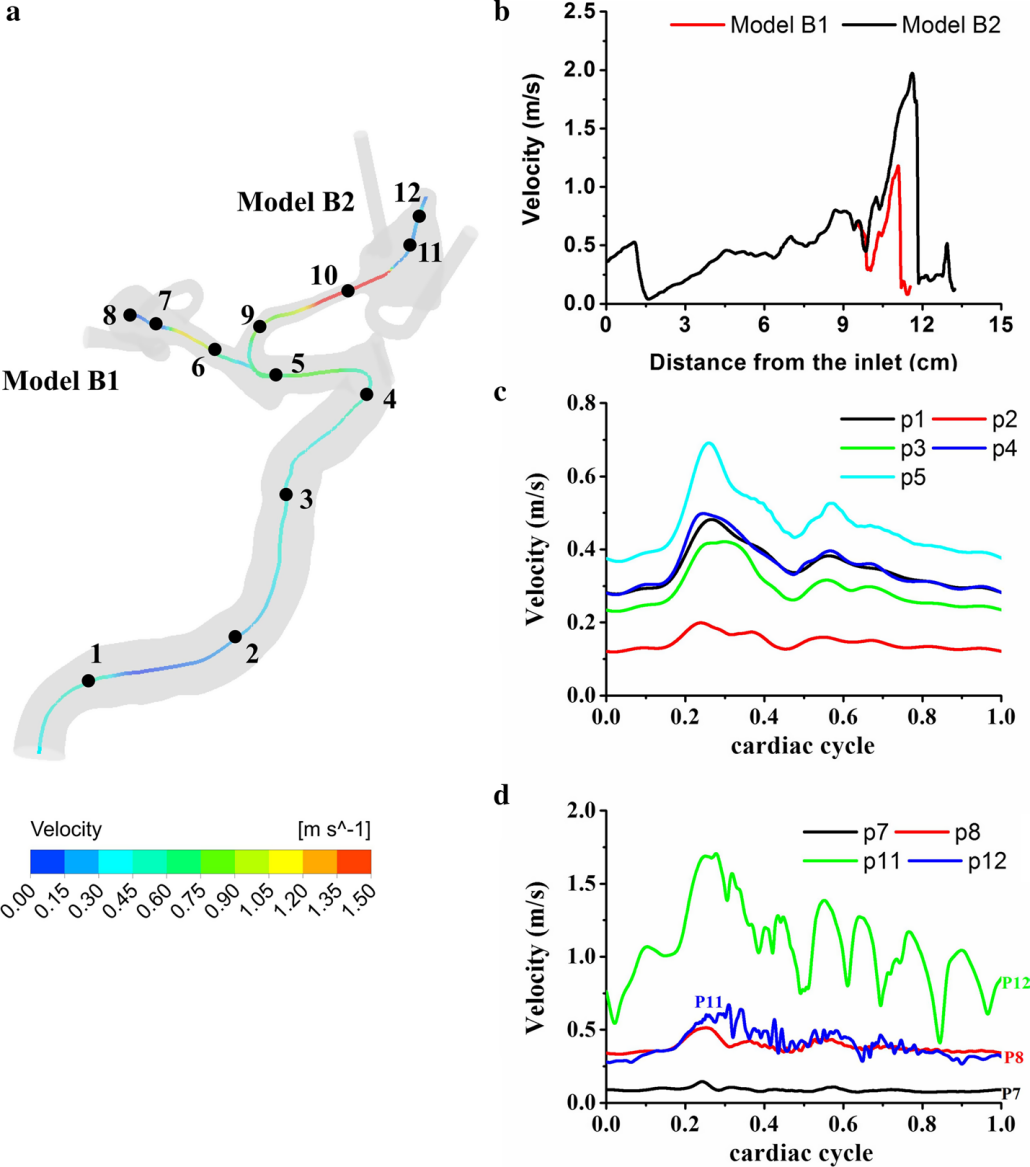
Velocity distributions aligning with centerlines at peak systole (**a**, **b**) and time-varying velocities at feature points for model* B* (**c**, **d**). Few inflection points and no velocity fluctuations are observed at the ICA siphon. The flow is very likely laminar and stable at ICA but does exhibit apparent fluctuations in the aneurysms. Note that the rupture aneurysm B2 (Points* 11*,* 12*) exhibit stronger velocity fluctuations compared with the unruptured aneurysm* B1* (Points* 7*,* 8*). The peak Reynolds numbers of the upstream parent arteries of aneurysms are calculated to be 718 and 746 for models* B1* and* B2*, respectively

**Fig. 7:**
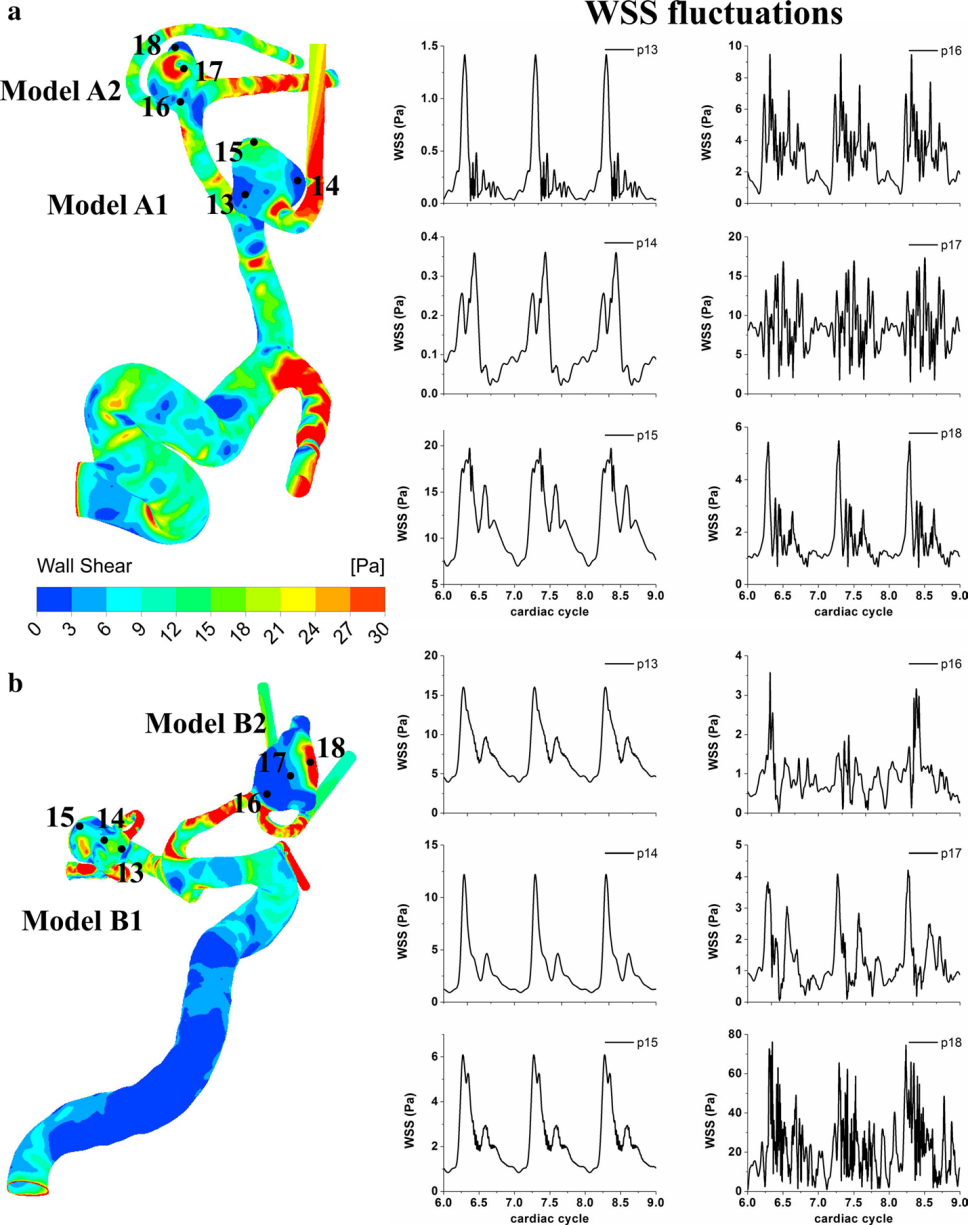
Time-varying WSSs at six feature points inside the cerebral aneurysms of model* A* (**a**) and model* B* (**b**). The ruptured aneurysms* A2* and* B2* apparently present stronger WSS fluctuations rather with the unruptured aneurysms* A1* and* B1* do

Figure 5a, b illustrates the velocity distributions aligning with centerlines at peak systole of cerebral aneurysms. Complicated velocity profiles with multiple inflection points are found at the ICA (Points 1–5). The velocity fluctuations originate from the bends of the ICA siphon and get developed in the upstream regions of aneurysms (Points 5–12). The ruptured aneurysm A2 (Points 11, 12) obviously presents more significant velocity fluctuations compared with the unruptured aneurysm A1 (Points 7, 8, Fig. 5d). On the other hand, model B shows few inflection points and almost no velocity fluctuations at the ICA (Fig. 6a–c). The flow in the vessels of model B is very likely laminar and stable at the ICA but does exhibit strong fluctuations in the aneurysms. Note that the ruptured aneurysm B2 (Points 11, 12) also show stronger velocity fluctuations compared with the unruptured aneurysm B1 (Points 7, 8). Figure 7a demonstrates the highly transient WSS behavior for models A1 and A2. Six feature points were located at the entrance of the aneurysm corresponding with the regions of WSS divergence. Minor WSS fluctuations are observed at the feature Points 13–15 in the unruptured aneurysm model A1 whereas the time traces of the feature Points 16–18 apparently demonstrate pronounced WSS fluctuations in the ruptured aneurysm model A2, which are present initially at peak systole when the flow turns to decelerate, being enhanced throughout the late systole. In Fig. 7b, it is further observed of obvious discrepancies of WSS fluctuations between model B1 (unruptured) and model B2 (ruptured): model B2 shows apparently intra-cycle WSS fluctuations rather than model B1 does.

**Fig. 8:**
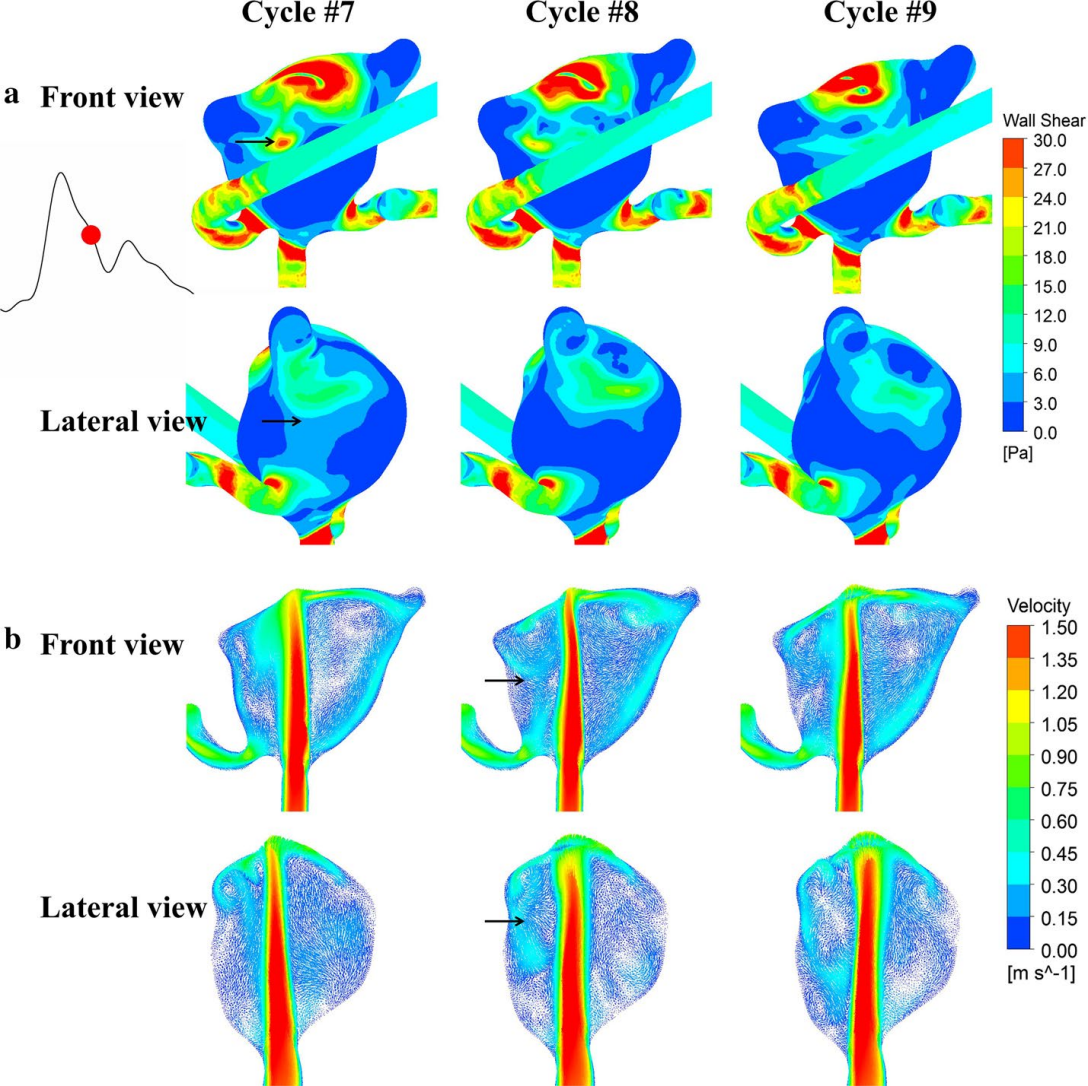
WSS distributions (**a**) and velocity fields in two cutting planes (**b**) at an instant of deceleration phase for three consecutive heart cycles (model* B2*). *Black arrows* point to the cycle-to-cycle differences of the flow patterns and WSS distributions during the deceleration phase

**Fig. 9:**
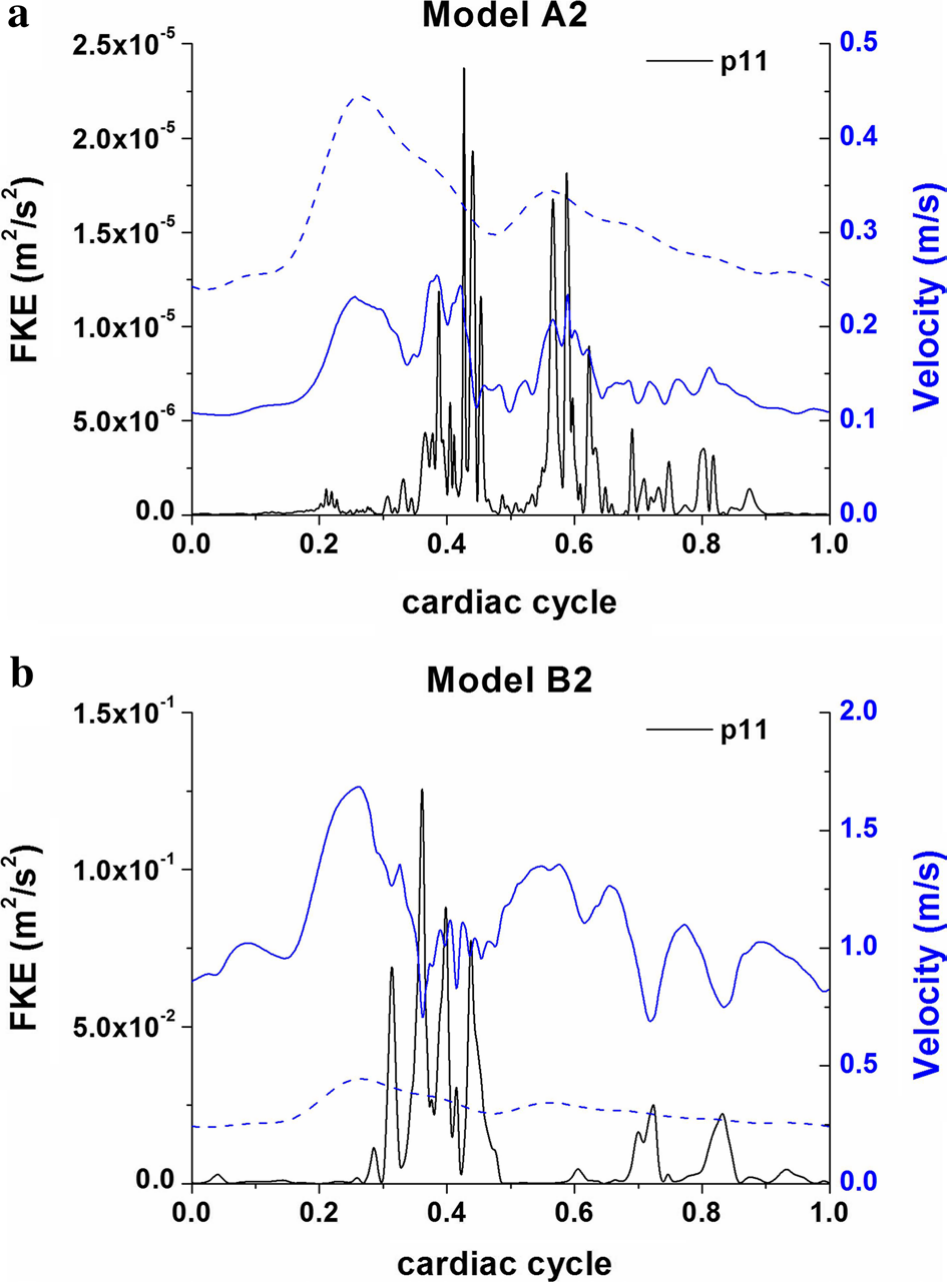
Average fluctuating kinetic energy (FKE) in model A2 (**a**) and model B2 (**b**). The *blue solid* and *dashed lines* show the time varying velocities at feature points inside aneurysms and inlets as reference, respectively. An obvious FKE peak is observed when flow turns to decelerate at late systole

Figure 8a indicates the instantaneous WSS distributions during the deceleration phase for three consecutive heart cycles of model B2. In the aneurysm model B2, cycle-to-cycle differences are already visible at peak systole, which substantially turn to be pronounced during the deceleration phase. Cycle-to-cycle fluctuations are slightly observed in the ruptured aneurysm A2. However, we see no differences in the unruptured aneurysms A1 and B1. Figure 8b illustrates the instantaneous velocity fields in two cutting planes of model B2. Different velocity distributions (black arrows) are observed in the aneurysm at late systole when the flow turns to decelerate. To quantify the cycle-to-cycle fluctuations, we further plot the averaged FKE measured at the feature points inside the aneurysms (Fig. 9). Note that the peak FKE value of model B2 is much larger than that of model A2 while the cycle-to-cycle fluctuations are confirmed in both the ruptured models B2 and A2. The flow in model A2 is apparently characterized by laminar flow until it turns to decelerate at late systole where the FKE reaches a peak of occurs 0.1–0.15 s (Fig. 9a). With Fig. 9b we further illustrate the time lag between the maximum FKE value and the maximum velocity and find that the peak FKE occurs at late systole in the ruptured aneurysm B2.

### Geometry and inflow waveform effects on flow fluctuations

**Fig. 10:**
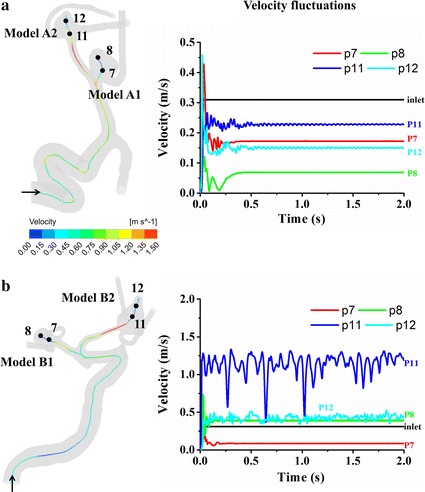
Time-varying velocities at feature points for model* A* (**a**) and model* B* (**b**). A constant inflow velocity of 0.31 m/s with no fluctuating component was imposed at the inlets to identify the possibility of flow instabilities independent of pulsatile flow dynamics. The rupture aneurysms* A2* and* B2* (Points* 11*,* 12*) exhibit consistent velocity fluctuations whereas unruptured aneurysms* A1* and * B1* (Points* 7*,* 8*) converge to a single solution

A constant inflow velocity of 0.31 m/s was imposed at the inlets of models A and B to investigate the geometry effects on the flow instabilities. Here we utilized a steady inflow boundary condition but solved the transient Navier–Stokes equations so as to identify the possibility of flow instabilities independent of pulsatile flow dynamics [17]. In Fig. 10, it is seen that the initial transient feature in velocity rapidly damps out in the unruptured aneurysm models A1 and B1 (Points 7, 8), whereas the ruptured aneurysm models A2 and B2 (Points 11, 12) demonstrates persistent velocity fluctuations. We further investigate whether the flow fluctuations arising in the upstream parent arteries (Figs. 5, 6) dominates the intra-cycle and inter-cycle flow fluctuations in cerebral aneurysms. An extensive study of CFD simulation was carried out with a truncated inlet instead of the segments of original ICA, which presents almost the same feature of velocity fluctuations in the ruptured aneurysm B2. Furthermore, we reduce the number of Fourier modes for the inflow waveform to investigate the impact of the waveform on the flow fluctuations. It was found that the flow instabilities initiated in the upstream region of aneurysms or the shape of the inlet waveform play a marginal role in the presence of the intra-aneurysm flow fluctuations.

## Discussion

With a unique patient-specific model of two matched-pairs of rupture-unruptured cerebral aneurysms we demonstrated that the two ruptured aneurysms apparently present stronger WSS fluctuations compared with the unruptured aneurysms in the same patient. Besides the intra-cycle fluctuations, it is interesting to find cycle-to-cycle fluctuations in the ruptured aneurysms when the flow turns to decelerate as reported in [14]. The obvious differences observed between the matched-pairs of ruptured–unruptured aneurysms imply that flow instabilities may be a potential source associated with the rupture mechanism of cerebral aneurysms.

### Geometry and inlet waveform effects on flow instabilities

Flow instability occurs under certain circumstances depending on geometrical configurations and inflow waveforms, which is inherently correlated with the existence of a primary adverse pressure gradient at late systole [21, 22]. With Figs. 5 and 6 we investigated whether there exists a correlation between flow fluctuations and aneurysm through the temporal velocities along the centerlines of vessels. The flow fluctuations of model A initially appeared at the ICA siphon, whereas model B exhibited flow fluctuations till the upstream region of MCA, pointing to the importance of the geometry-dependence of the flow instabilities. We then prescribed a uniform inflow velocity with no fluctuating component at the inlets and found that velocity fluctuations still exist in the ruptured aneurysms A2 and B2 rather than the unruptured aneurysms A1 and B1 (Fig. 10). On the other hand, inlet flow rate waveforms could also lead to the flow instability associated with cerebral aneurysms. We furthered investigated whether the high-frequency flow fluctuations arising in the upstream parent arteries of aneurysms influence the flow fluctuations in cerebral aneurysms and observed that its influence is marginal. This support a conclusion reached in our previous study that the low-frequency harmonics very likely induce and dominate WSS fluctuations associated with the cerebral aneurysms, in particular during the decelerating phase at late systole, which corresponds with a steeper global and local pressure gradient [22].

### Implications of WSS fluctuations

Studies of endothelial cells (ECs) associated with aneurysms could help to understand the potential association between WSS fluctuations and the aneurysm rupture. The pathological differences were observed between the ruptured and unruptured cerebral aneurysms [23–25]. Kataoka et al. [23] studied 44 ruptured and 27 unruptured aneurysms and observed that the ECs in ruptured aneurysms were most likely damaged with disrupted arrangements, whereas most unruptured aneurysms were covered with normally shaped arterial ECs. Frösen et al. [24, 25] also found that the ruptured aneurysms were characterized by loss of ECs. The ruptured aneurysms manifested obvious endothelial damage and wall degeneration, which correlated to the oscillatory shear stress. The response of ECs exposed to shear stress has been studied by varying the frequency, direction and gradient [26, 27]. Chappell et al. [26] proposed that the oscillatory shear stress in vitro provided a proinflammatory stimulus to human ECs and suggested the ECs in vivo might be greatly affected by oscillatory shear stress. Himburg et al. [27] investigated the frequency-dependent response of the vascular endothelium to pulsatile shear stress and observed that the proinflammatory response evoked by the higher frequency was most pronounced under reversing and oscillatory shear. These studies in the field of cell biomechanics and mechanobiology provide evidence that that the high-frequency oscillation/fluctuation and rapidly spatial/gradient variation but not the absolute magnitude of WSSs, could have more impact on the life cycle of the aneurysm.

From the viewpoint of energy loss, the WSS fluctuations with pronounced energy content can introduce extra friction and transfer substantial energy to the flow fluctuations. Several studies have demonstrated the significant association between aneurysm rupture with energy/pressure losses [28, 29], which inherently correlated to flow instabilities [21, 22]. These results imply that WSS fluctuations may play an important role in aneurysm rupture associated with the energy/pressure loss. The potential associations between WSS fluctuations and aneurysm rupture still need to be further studied in the future.

### Potential limitations

The boundary conditions employed in the present study are not patient-specific and the effects of waveform reflections at outlets were not taken into consideration. The compliance effects of vessel and aneurysm on the flow instability are not considered because the clinical data of pressure waves and wall thicknesses were not available for the two models, which may to some extent influence the flow patterns and hence the flow fluctuations in the cerebral aneurysm models [30, 31]. In addition, the non-Newtonian feature of blood flow may not be negligible in highly disturbed flows associated with cerebral aneurysms, though it is normally assumed to be less important in medium and large arteries in laminar flows due to its modest effect on flow patterns [32, 33]. Obviously, effects of both viscoelastic behavior of vessel/aneurysm walls and non-Newtonian characteristics of highly fluctuated flows associated with aneurysms on the flow instabilities as well as its association with aneurysm rupture remain unknown yet and still need further study. Although the signs of fully developed turbulence are not detected clearly in the cerebral aneurysms, the flow fields associated with the ruptured aneurysms present highly disturbed flows near a transition point, which may lead to a transition from laminar to turbulence even at such low Reynolds numbers of several hundreds. This is out of the scope of the present study but needs to be further investigated in the future.

## Conclusions

The present study investigated the hemodynamic factors and flow instabilities in two patients with multiple intracranial aneurysms. Our results demonstrated highly disturbed flows in the ruptured aneurysms, which are characterized by pronounced velocity and WSS fluctuations at systole. The two ruptured aneurysms experienced apparently temporal intra-cycle and inter-cycle WSS fluctuations compared with the two unruptured aneurysms of the same patient, which suggests that flow instability may be a potential source relating to the rupture of multiple aneurysms.

## Abbreviations

CFD: computational fluid dynamics
MCA: middle cerebral artery
ACA: anterior communicating artery
WSS: wall shear stress
OSI: oscillatory shear index
FKE: fluctuating kinetic energy
SAH: subarachnoid hemorrhage
ECs: endothelial cells
